# Extremes of Liver Transplantation for Hepatocellular Carcinoma

**DOI:** 10.3390/jcm8060787

**Published:** 2019-06-03

**Authors:** Michał Grąt, Maciej Krasnodębski, Marek Krawczyk, Jan Stypułkowski, Marcin Morawski, Michał Wasilewicz, Zbigniew Lewandowski, Karolina Grąt, Waldemar Patkowski, Krzysztof Zieniewicz

**Affiliations:** 1 Department of General, Transplant and Liver Surgery, Medical University of Warsaw, 1A Banacha Street, 02-091 Warsaw, Poland; michal.grat@gmail.com (M.G.); marek.krawczyk@wum.edu.pl (M.K.); jan.styp@gmail.com (J.S.); marcin.b.morawski@gmail.com (M.M.); waldemar.patkowski@gmail.com (W.P.); krzysztof.zieniewicz@wum.edu.pl (K.Z.); 2 Liver and Internal Medicine Unit, Department of General, Transplant and Liver Surgery, Medical University of Warsaw, 02-091 Warsaw, Poland; newdodo@poczta.onet.pl; 3 Department of Epidemiology and Biostatistics, Medical University of Warsaw, 3 Oczki Street, 02-007 Warsaw, Poland; zbigniew.lewandowski@wum.edu.pl; 4 Second Department of Clinical Radiology, Medical University of Warsaw, 02-091 Warsaw, Poland; karolina.grat@gmail.com

**Keywords:** hepatocellular carcinoma, liver transplantation, alpha-fetoprotein, tumor recurrence

## Abstract

The aim of this retrospective observational study was to evaluate outcomes of patients with extremely advanced hepatocellular carcinoma (HCC) after liver transplantation. A total of 285 HCC patients after liver transplantation were screened for eligibility based on either intrahepatic dissemination (≥10 tumors) or macrovascular invasion. Tumor recurrence was the primary end-point. The study cohort comprised 26 patients. Median recurrence-free survival was 23.2 months with hepatitis B virus (HBV) infection (*p* = 0.038), higher AFP model score (*p* = 0.001), prolonged graft ischemia (*p* = 0.004), and younger donor age (*p* = 0.016) being significant risk factors. Median recurrence-free survival of HBV-negative and HBV-positive patients was 29.8 and 9.3 months, respectively (*p* = 0.053). In patients with macrovascular invasion, recurrence-free survival at 3 years was 46.3% with no specific predictors. Tumor size (*p* = 0.044), higher AFP model score (*p* = 0.019), prolonged graft ischemia (*p* = 0.016), and younger donor age (*p* = 0.041) were significant risk factors in patients with intrahepatic dissemination. Superior 3-year outcomes were observed in patients with intrahepatic dissemination and tumor size <3.5 cm (83.3%, *p* = 0.027) and HBV-negative patients with ischemia <9.7 h (85.7%, *p* = 0.028). In conclusion, patients with extremely advanced HCCs are remarkably heterogeneous with respect to their profile of tumor recurrence risk. This heterogeneity is largely driven by factors other than standard predictors of post-transplant HCC recurrence.

## 1. Introduction

The incidence rates of hepatocellular carcinoma (HCC) are increasing worldwide, along with the associated economic and organizational burden to healthcare systems [1,2,3]. Progress in diagnosis and management of patients has led to continuous improvements of survival outcomes [1,4]. Diagnosis of patients with HCCs at early stages is particularly important, as these are more likely to undergo liver resection or transplantation, the two potentially radical treatment modalities associated with clearly superior survival rates [5]. Accordingly, median survival of patients is highly dependent on disease stage at diagnosis [5,6]. However, majority of HCCs are diagnosed at or beyond intermediate stage with excessive tumor burden often precluding the use of potentially radical strategies [5,6,7]. Despite progress in the field of palliative therapies, survival of patients with advanced HCCs remains poor. For patients with advanced and terminal stage HCCs, median survival does not exceed several months [5]. Notably, utilization of aggressive surgical treatment for patients with advanced tumors was reported to improve the negative prognoses, which was inconsistent with Barcelona Clinic Liver Cancer (BCLC) guidelines, pointing to the need for more personalized approaches [8,9].

Liver transplantation not only can be used in patients with unresectable tumors but also offers a clear survival advantage over resection, yet its availability is highly limited by the donor numbers [10,11]. Nevertheless, expansion of the conservative Milan criteria is currently in progress, most frequently on the basis of combining morphological and biological features, such as alpha-fetoprotein concentration, tumor differentiation, inflammatory markers, and response to neoadjuvant therapies [12,13,14,15,16,17]. However, the majority of the current proposals widen the selection criteria moderately and still do not extend towards patients with the most advanced tumors. Notably, contrary to Barcelona Clinic Liver Cancer (BCLC) guidelines, increasing stages have been reported to favor survival benefit [18]. Although this does not directly imply the superior survival benefit of patients with excessive tumor burden, as BCLC stage is also dependent on non-tumor related factors, higher number of tumors, increased tumor diameter, and unfulfillment of Milan criteria have recently been reported as positive predictors of survival benefit after liver transplantation for HCC [19]. However, these results were obtained using statistical modelling rather than real-life data, and thus are potentially subject to major selection bias. Therefore, these results seem insufficient to consider wide broadening of selection criteria for patients with advanced HCCs confined to the liver in the context of potential harm to non-HCC patients on the waiting lists and unacceptable survival outcomes observed for such patients in the past decades [20]. The aim of this study was to evaluate the outcomes after liver transplantation for HCC in patients with the most advanced tumors, defined by intrahepatic dissemination or macroscopic vascular invasion.

## 2. Experimental Section

This was a retrospective observational study. Inclusion criteria were as follows: at least 10 HCC lesions in the explanted liver confirmed in the pathological report, or in the case of fewer tumors, presence of macroscopic vascular invasion in pathological reports. Tumor number of 10 was chosen as it is the 95th percentile for tumor number in all HCC liver transplants in our Department and the upper border for number of lesions to report in explant pathological reports. Exclusion criteria were: fibrolamellar HCCs, combined hepatocellular/cholangiocellular cancer, and carcinosarcoma. A total of 285 HCC patients after liver transplantation in the Department of General, Transplant, and Liver Surgery at the Medical University of Warsaw (Poland) in the period between January 2001 and April 2017 were screened for eligibility. Following application of the inclusion criteria, the study cohort comprised 26 patients. The study protocol was approved by the institutional review board of the Medical University of Warsaw. Informed consent was not obtained from the patients due to the retrospective nature of the study, which is in line with institutional review board and national regulations. All methods were performed in accordance with the relevant guidelines and regulations. No organs were procured from prisoners. All organs were procured by the transplant team of the Department of General, Transplant, and Liver Surgery of the Medical University of Warsaw.

Tumor recurrence was the primary end-point of the study. It was used to calculate recurrence-free survival, defined as time from liver transplantation until HCC recurrence and censored on the date of last available follow-up or death for non-HCC related causes, whichever occurred first. Details on perioperative management, immunosuppressive treatment, and follow-up protocol were provided previously [21,22]. Generally, patient selection process was based on the fulfillment of criteria of either the Up-to-7 or University of California, San Francisco, with additional fetoprotein cut-off of 100 ng/mL, which has been utilized since 2013 (the Warsaw criteria) [15]. Nevertheless, patients with more excessive tumor burden were also selected at the discretion of the multidisciplinary tumor board.

First, recurrence-free survival was compared depending on the inclusion criteria. Second, risk factors for HCC recurrence were analyzed in all patients. Finally, subgroup analyses were performed following division of patients based on inclusion criteria and established risk factors for HCC recurrence.

Qualitative and quantitative data were presented as numbers with frequencies and medians with interquartile ranges (IQRs), respectively. Survival estimations were based on the Kaplan-Meier method. Comparisons of survival curves were done with log-rank test. Reversed Kaplan-Meier estimator was applied to calculate median follow-up. Cox proportional hazards regression was used for analyses of risk factors for tumor recurrence. Receiver operating characteristics (ROC) curves were analyzed to identify optimal cut-offs of quantitative variables in prediction of HCC recurrence. Hazard ratios (HRs) and c-statistics were presented with 95% confidence intervals (95% CI). Two-sided *p* < 0.05 was considered significant. Analyses were computed using STATISTICA version 13.1 software (Dell Inc., Tulsa, OK, USA).

## 3. Results

Basic patient, donor, and operative characteristics for all 26 liver transplant recipients included in the study cohort, 20 patients with intrahepatic HCC dissemination, and 10 with macrovascular invasion are presented in Table 1. Considering the inclusion criteria, 16 patients had intrahepatic dissemination, 6 patients had macrovascular invasion, and 4 patients had both intrahepatic dissemination and macrovascular invasion. Macrovascular invasion was missed on preoperative imaging in all except 1 patient, in whom thrombosis of middle hepatic vein was misclassified as benign on computed tomography. Median interval between preoperative imaging and transplantation in these patients was 2.3 months and mean waiting time was 2 months. Median duration of follow-up was 22.6 months (IQR: 9.2–36.7). A total of 12 patients developed HCC recurrence following a median of 11.4 months (IQR: 5.1–22.1).

**Figure 1:**
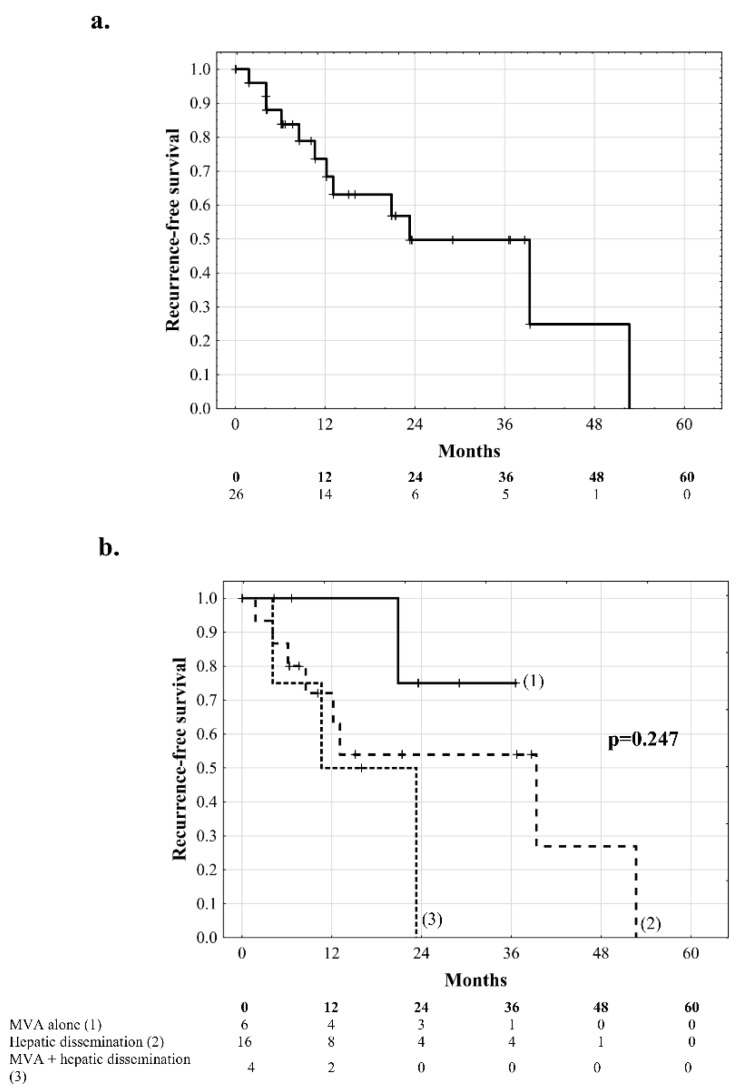
Recurrence-free survival of (**a**) all patients included in the study and (**b**) comparison between patients with macrovascular invasion (MVA) alone (**1**), hepatic dissemination alone (**2**), and both macrovascular invasion and intrahepatic dissemination (**3**). Numbers of patients at risk are presented below the graphs.

Median recurrence-free survival of all patients included in the study was 23.2 months (IQR: 10.1–39.3 months), with 1, 2, 3, and 5 year rates of 73.6%, 49.7%, 49.7% and 0.0%, respectively (Figure 1a). Patients with macrovascular invasion exhibited a 3-year recurrence-free survival of 75.0%, patients with hepatic dissemination without macrovascular invasion exhibited 3-year and 5-year recurrence-free survival of 54.0% and 0.0%, respectively, with a median of 16.0 months (IQR: 7.6–40.4 months), and the corresponding values for patients with hepatic dissemination and macrovascular invasion were 0.0% at 2 years and 10.6 months, respectively (*p* = 0.247, Figure 1b). Risk factors for tumor recurrence in all patients included presence of hepatitis B virus (HBV) infection (*p* = 0.038), higher AFP model score (*p* = 0.001), longer total duration of graft ischemia (*p* = 0.004), and younger donor age (*p* = 0.016), while standard HCC-related factors were not significantly associated with the risk of tumor recurrence (Table 2).

**Figure 2:**
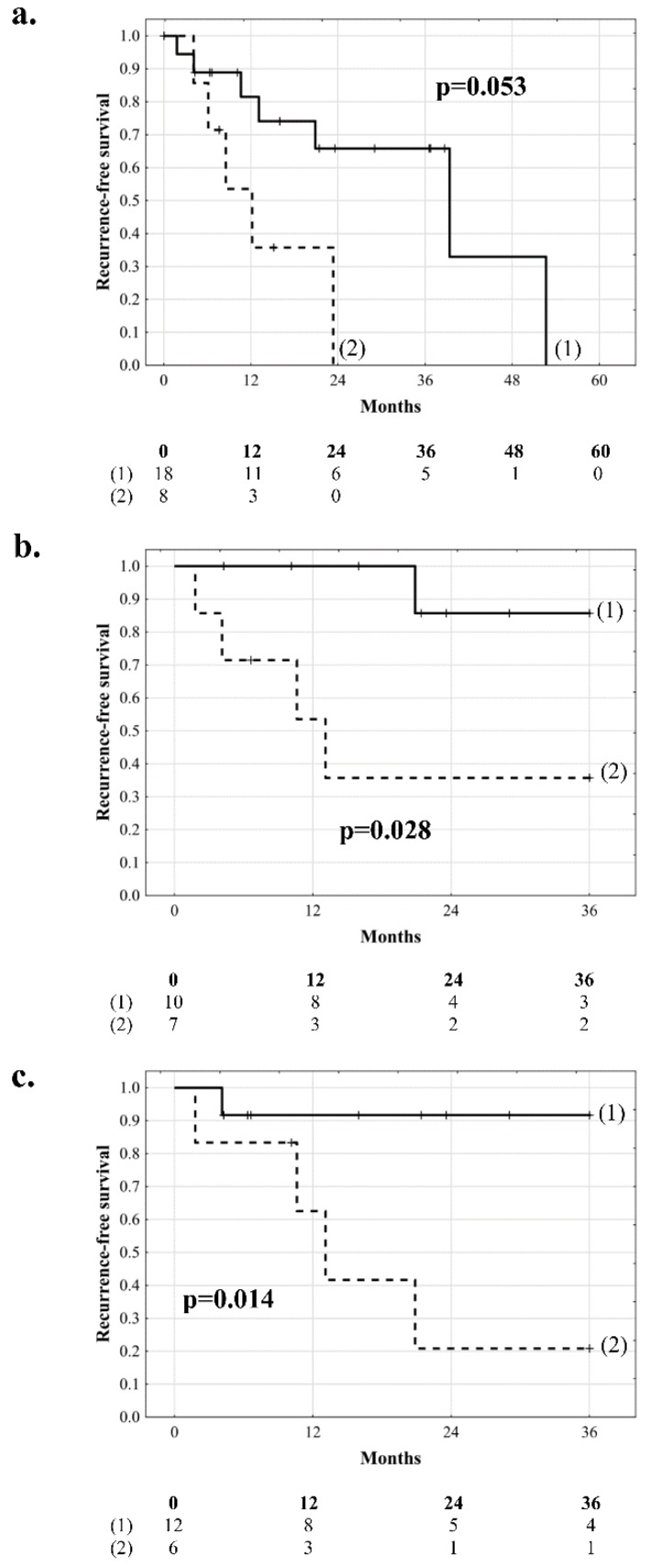
Recurrence-free survival comparisons between (**a**) patients without (**1**) and with (**2**) hepatitis B virus infection, (**b**) patients without hepatitis B virus infection and duration of graft ischemia <9.7 h (**1**) and ≥9.7 h (**2**), and (**c**) hepatitis B virus-negative recipients of grafts procured from donors >47 years (**1**) and ≤47 years (**2**) of age. Numbers of patients at risk are presented below the graphs.

HBV-positive patients and HBV-negative patients exhibited median recurrence-free survival of 9.3 months (IQR: 5.6–15.5) and 29.8 months (IQR: 12.8–42.6), respectively, (*p* = 0.053), with recurrence-free survival rates reaching 0% after 1.9 years and 4.4 years, respectively (Figure 2a). In HBV-negative patients, risk factors for tumor recurrence comprised longer total duration of graft ischemia (*p* = 0.016), higher AFP model score (*p* = 0.004), and younger donor age (*p* = 0.017, Table 3). Optimal cut-offs in prediction of HCC recurrence were ≥9.7 h for total ischemia (c-statistic: 0.840, 95% CI 0.671–1.000) and ≤47 years for donor age (c-statistic: 0.750, 95% CI 0.562–0.938). Recurrence-free survival of patients without HBV infection and total duration of graft ischemia <9.7 h and ≥9.7 h at 3 years was 85.7% and 35.7%, respectively (*p* = 0.028, Figure 2b). The corresponding recurrence-free survival rates of HBV-negative recipients of grafts procured from donors aged >47 years and ≤47 years were 91.7% and 20.8% (median: 12.1 months, IQR: 5.3–19.3), respectively (*p* = 0.014, Figure 2c).

**Figure 3:**
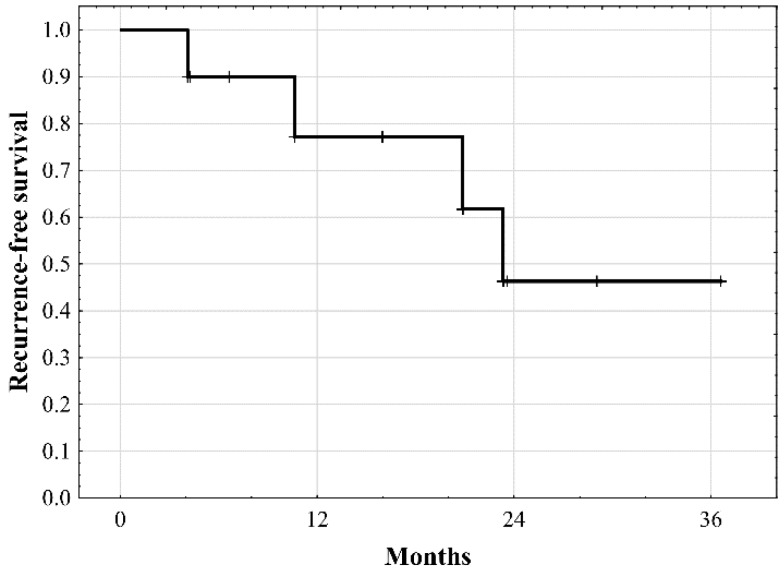
Recurrence-free survival of patients with macrovascular invasion undergoing liver transplantation.

In the subgroup of 10 patients with macrovascular invasion, median recurrence-free survival was 22.7 months, with the rates of 77.1% at 1 year and 46.3% at 2 and 3 years (Figure 3). No specific risk factors for tumor recurrence were identified in this cohort of patients (Table 4).

**Figure 4:**
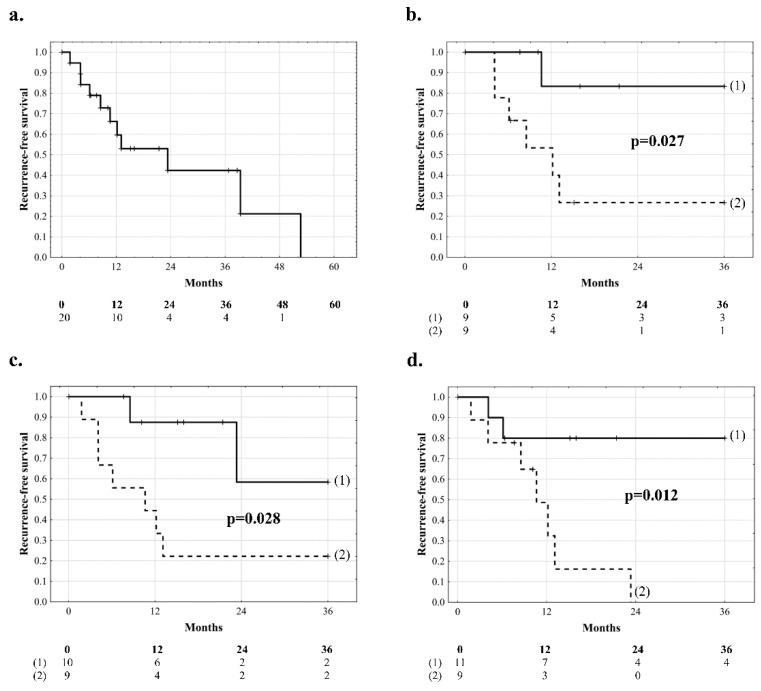
Recurrence-free survival of patients with intrahepatic dissemination (**a**) and comparisons between patients with intrahepatic dissemination and (**b**) tumors <3.5 cm (**1**) and ≥3.5 cm (**2**), (**c**) total duration of graft ischemia of <9.7 h (**1**) and ≥9.7 h (**2**), and (**d**) receiving grafts from donors aged >47 years (**1**) and ≤47 years (**2**). Numbers of patients at risk are presented below the graphs.

In the subgroup of 20 patients with intrahepatic tumor dissemination, median recurrence-free survival was 16.0 months (IQR: 7.7–36.5) with the rates of 66.2% at 1 year, 42.4% at 2 years, 21.2% at 4 years, and 0.0% at 4.4 years (Figure 4a). In these patients, risk factors for tumor recurrence comprised larger size of the largest tumor on explant pathology (*p* = 0.044), higher AFP model score (*p* = 0.019), longer total duration of graft ischemia (*p* = 0.016), and younger donor age (*p* = 0.041, Table 3). Optimal cut-offs for prediction of tumor recurrence were ≥ 3.5 cm for tumor size on explant pathology (c-statistic: 0.753, 95% CI 0.520–0.986; *p* = 0.033), ≥9.7 h for total ischemia (c-statistic: 0.903, 95% CI 0.748–1.000; *p* < 0.001), and ≤47 years for donor age (c-statistic: 0.677, 95% CI 0.437–0.917; *p* = 0.149). Recurrence-free survival at 3 years in patients with intrahepatic dissemination was 83.3% and 26.7% for those with tumors <3.5 cm and ≥3.5 cm on explant pathology, respectively (*p* = 0.027, Figure 4b), 58.3% and 22.2% when total duration of graft ischemia was <9.7 h and ≥9.7 h, respectively (*p* = 0.028, Figure 4c), and 80.0% and 0.0% (23.3 months) with median of 10.5 months (IQR: 5.1–12.6) for recipients of grafts from donors aged >47 years and ≤47 years, respectively (*p* = 0.012, Figure 4d). Recurrence-free survival in patients with AFP model under 2 points was 0% after 39 months post-transplantation, as compared to 0% at 53 months with AFP models over 2 points in the entire study cohort (*p* = 0.182), and in sub-groups of patients without HBV infection (*p* = 0.704) and with intrahepatic dissemination (*p* = 0.121).

## 4. Discussion

Liberal selection of HCC patients for liver transplantation has historically resulted in extremely poor outcomes related to very high risk of tumor recurrence [20]. The introduction of the Milan criteria changed the landscape of liver transplantation for HCC and dramatically limited this risk, and thus, has driven the selection process for years [23]. Increasing knowledge on the relevance of factors other than morphological criteria with respect to prediction of HCC recurrence, such as alpha-fetoprotein concentration, inflammatory markers, and response to neoadjuvant therapies, has enabled changes in selection policy towards moderate liberalization [12,13,14,15,16,17]. Reports based on both equity and survival benefit approaches indicate that selected patients with even more advanced tumors should not always be deprived of the chances of transplantation [18,19,24]. However, the majority of studies are based on an overwhelming proportion of patients meeting or only moderately exceeding Milan criteria [12,13,14,16]. The results of the present study provide unique data on outcomes and risk factors for tumor recurrence in a population of patients with the most advanced HCCs, who initially are at extremely high risk of tumor recurrence. Despite risk heterogeneity, the observed outcomes do not justify the use of scarce resources of donor organs, neither in patients with intrahepatic dissemination nor in those with macrovascular invasion.

Overall, the study indicates that patients with the most advanced tumors universally develop recurrence with a median recurrence-free survival of less than 2 years. However, the median overall survival of patients treated with systemic therapy or radioembolization, which seem to be the only realistic alternatives, does not currently exceed one year [25]. Accordingly, these patients can undoubtedly benefit from undergoing liver transplantation, yet not at a magnitude justifying the use of scarce resources of donor organs. The use of liver transplantation has already been reported as a preferable alternative to palliative therapies for advanced HCC patients, however still with remarkably lower tumor burden than in patients included in the present study [26]. Notably, 42% (11 of 26) of patients had an AFP model of 2 and less points in the whole studied cohort, potentially enabling liver transplantation according to this proposal. From this group, 38% (6 of 16) of patients with intrahepatic dissemination and no macrovascular invasion also had an AFP Model of at most 2 points. The model was also confirmed as a risk factor for tumor recurrence in all patients and in the subgroup of patients without hepatitis B virus infection and with intrahepatic tumor dissemination. However, obtained results of patients’ survival do not confirm their transplant eligibility, despite having AFP model scores of 2 and less. While patients beyond Milan criteria but with AFP models of up to 2 points were recently shown to be at an increased, yet still acceptable, risk of tumor recurrence, this does not seem to apply to patients with intrahepatic dissemination [27]. Moreover, our outcomes could be a reason to discuss if the AFP model needs an upper limit of tumors in its own classification score.

Although this study generally provides evidence for extreme caution in potential development of strategies of wide liberalization of selection criteria in the context of transplant utility, the results point towards remarkable heterogeneity of the recurrence risk, even in patients with extremely advanced HCCs. Notably, there were no significant differences in outcomes related to the presence of macrovascular invasion or intrahepatic tumor dissemination, and neither the latter nor tumor number had a significant impact on the risk of recurrence in the whole cohort. Patients with macrovascular invasion exhibited uniformly unacceptable outcomes, with no identified specific risk factors for HCC recurrence and less than 50% recurrence-free survival at 3 years, precluding the use of liver transplantation in this population. These results are in line with the well-known major association between presence of macrovascular invasion and the risk of post-transplant HCC recurrence, even in cases of low alpha-fetoprotein concentration [28,29]. Notably, Lee at al. advocated not considering macrovascular invasion as an absolute contraindication for transplantation in their recent report based on 10 patients, despite 45.5% 3-year recurrence-free survival, almost identical to that observed in the present study [30]. The authors suggested using alpha-fetoprotein concentration and tumor size, among other criteria, as useful factors in selection of transplant-eligible patients with macrovascular invasion. As neither of the two factors was a significant predictor of HCC recurrence in this population, the present study does not confirm the previous findings and point towards considering macrovascular invasion as an absolute contraindication. Scarce reports on the effectiveness of pre-transplant radioembolization need further confirmation [31,32]. Therefore, according to the presented results, patients with macrovascular invasion should not be selected for liver transplantation.

Overall, HBV-negative patients with advanced HCCs exhibited more than 3-fold longer median recurrence-free survival as compared to HBV-positive patients. This finding is contrary to previous observations of particularly high risk of post-transplant tumor recurrence in patients with advanced HCCs associated with hepatitis C virus rather than HBV [33]. In general, the majority of studies point towards superior outcomes of HCC patients associated with the presence of HBV [34,35,36]. The reasons for this discrepancy may include a completely different population of patients, yet remains not completely understood. Nevertheless, the present study indicates that HBV-related extremely advanced HCCs are characterized by increased biological aggressiveness. However, from the transplant perspective, higher median recurrence-free survival does not advocate liver transplantation for HBV-negative, very high-risk HCC patients, since the problem of tumor recurrence is also universal in this population.

The only “standard” risk factor for post-transplant HCC recurrence identified in the present study was size of the largest tumor in a subgroup of patients with intrahepatic dissemination. Although numerous previous studies identified this factor as a major predictor of recurrence, the negative effects of increasing tumor diameter on the results of liver transplantation for HCC in patients with tumor burden of such extent are quite surprising [12]. As the 3-year recurrence-free survival for patients with tumors below 3.5 cm exceed 80%, this may be a population in which further predictors of recurrence should be searched for in potential future studies focused on transplant eligibility in cases of extremely advanced HCCs. Provided that pre-transplant alpha-fetoprotein concentration does not exceed 100 ng/mL, the majority of these patients do fulfill the AFP-Model-based cut-off for transplant eligibility of up to 2 points [12]. Nevertheless, the results are insufficient to indicate which patients with numerous small tumors are potentially at acceptable risk of tumor recurrence from the transplant eligibility perspective, as the alpha-fetoprotein concentration did not emerge as a significant risk factor.

Prolonged graft ischemia and younger donor age were uniformly identified as risk factors for HCC recurrence, both in all patients and in subgroups with intrahepatic dissemination or without HBV infection. Negative effects of the former are most probably related to increased extent of ischemia-reperfusion injury, a phenomenon potentially inducing the risk of metastases formation [37,38,39]. This is consistent with previous reports on the association between ischemic times and the risk of post-transplant HCC recurrence, particularly in high-risk individuals, namely those with vascular invasion or ^18^F-fluoro-deoxy-glucose avid tumors, on positron emission tomography [40,41]. Considering the association between younger donor age and increased risk of recurrence, there seems to be no clear explanation. Given the increased susceptibility of grafts obtained from older donors to ischemia-reperfusion injury and its negative consequences, an opposite association was expected. In fact, it is the older donor age that was previously identified as a risk factor for post-transplant HCC recurrence [42]. The only previously identified negative impact of younger donor age was recently reported in a study on living donor liver transplantation for HCC, in which a negative effect of male sex of the donor limited to recipients of grafts from donors younger than 40 years was observed [43]. However, it is highly unlikely that this is responsible for the observed negative impact of younger donor age, as neither donor sex was a risk factor for HCC recurrence, nor there were significant differences in the distribution of donor sex according to donor age (data not shown). Finally, the two variables related to graft quality are of little or no importance in evaluating transplant eligibility.

This study has several limitations beyond those inherent to its retrospective nature. First, it is limited by the number of patients, which is a natural consequence of inclusion of only those with extremely advanced HCCs. Nevertheless, considering current selection policies, obtaining a larger population for potential analyses is rather unlikely, yet the present study points toward a multi-center analysis. Such a multi-institutional analysis may add more insight into predictors of recurrence in this unique population. Second, the estimated recurrence-free survival rates may be biased by non-HCC related deaths, however the numbers precluded the use of competing risk models. Despite the liberal selection policy, the study cohort may be not fully representative for the entire population of patients with intrahepatic HCC dissemination or macrovascular invasion. Finally, the cut-off for tumor number of 10 for defining intra-hepatic dissemination for the purpose of this study might be misleading, as majority of these patients had uncountable tumors. However, 10 was the highest number of reports in histopathological examinations, and thus we were not able to better characterize this population. Given that almost all patients had only one instance of preoperative imaging, it was impossible to evaluate pretransplant progression, which could be an important prognostic factor.

In conclusion, the present study provides unique up-to-date data on the outcomes of patients with most advanced HCCs undergoing liver transplantation. This population seems highly heterogeneous with respect to HCC recurrence risk. Both macrovascular invasion and intrahepatic tumor dissemination should remain contraindications for liver transplantation, yet potential further studies on wide liberalization of transplant eligibility criteria should focus on patients with numerous small HCCs. In patients with extremely advanced HCCs, HBV infection and prolonged graft ischemia are major risk factors for post-transplant recurrence. Interestingly, pre-transplant alpha-fetoprotein concentration does not seem to have prognostic relevance in this population.

## Figures and Tables

**Table 1:** Characteristics of all 26 patients after liver transplantation for hepatocellular carcinoma included in the study cohort, including 20 patients with intrahepatic tumor dissemination and 10 patients with macrovascular invasion.

Factors	All Patients (*n* = 26)	Intrahepatic Dissemination (*n* = 20)	Macrovascular Invasion (*n* = 10)
Sex			
male	21 (80.8%)	16 (80.0%)	7 (70.0%)
female	5 (19.2%)	4 (20.0%)	3 (30.0%)
Age (years)	58 (53–61)	57 (53–61)	57 (52–61)
Model for end-stage liver disease	11 (8–16)	10 (8–16)	11 (8–16)
Hepatitis B virus infection	8 (30.8%)	8 (40.0%)	2 (20.0%)
Hepatitis C virus infection	18 (69.2%)	14 (70.0%)	7 (70.0%)
Alcoholic liver disease	5 (19.2%)	3 (15.0%)	2 (20.0%)
AFP model	3 (2–5)	3 (2–5)	3 (1–5)
Tumor number	– (≥10) ^a^	– (≥10) ^a^	3.5 (1–>10)
Size of the largest tumor (cm)	3.7 (2.6–5.2)	3.4 (2.5–5.0)	5.0 (2.5–5.0)
Alpha-fetoprotein concentration (ng/mL)	24 (6–560)	14 (3–571)	167 (25–560)
Ca 19-9 concentration (U/mL)	41 (21–47)	39 (16–47)	41 (35–45)
Poor tumor differentiation	6 (23.1%)	5 (25.0%)	3 (30.0%)
Microvascular invasion	16 (64.0%)	10 (52.6%)	9 (90.0%)
Macrovascular invasion	10 (38.5%)	4 (20.0%)	10 (100.0%)
Neoadjuvant treatment	10 (38.5%)	7 (35.0%)	4 (40.0%)
Total ischemic time (hours)	9.5 (7.8–10.4)	9.6 (8.5–10.5)	8.6 (7.3–10.0)
Donor age	51 (38–54)	49 (37–53)	50 (43–61)
Donor sex			
male	13 (50.0%)	12 (60.0%)	5 (50.0%)
female	13 (50.0%)	8 (40.0%)	5 (50.0%)
Intraoperative PRBC transfusions (units)	3.5 (2–5.5)	4 (2–6)	4 (2–5)
Intraoperative FFP transfusions (units)	6 (4.5–10.0)	6 (4–10)	8 (5–10)

Data are presented as number (%) or median (interquartile range); ^a^ = not calculated, as majority of tumors were classified as uncountable or numerous. Ca 19-9 PRBC = packed red blood cells; FFP = fresh frozen plasma.

**Table 2:** Analyses of risk factors for tumor recurrence after liver transplantation in all 26 patients with intrahepatic tumor dissemination and macrovascular invasion included in the study.

Factors	HR (95% CI)	*p*
Male sex	0.62 (0.12–3.15)	0.560
Age	0.99 (0.91–1.09)	0.886
MELD	1.04 (0.94–1.14)	0.437
HBV infection	3.84 (1.08–13.64)	0.038
HCV infection	0.70 (0.18–2.83)	0.622
ALD	1.02 (0.26–3.93)	0.979
AFP model	1.45 (1.09–1.94)	0.001
Tumor number	1.15 (0.89–1.48)	0.274
Tumor size	1.40 (0.92–2.11)	0.113
Alpha-fetoprotein	1.06 (0.87–1.29)	0.552
Ca 19-9	0.83 (0.32–2.19)	0.710
Poor tumor differentiation	4.09 (0.90–18.47)	0.067
Microvascular invasion	0.58 (0.16–2.12)	0.414
Macrovascular invasion	1.02 (0.27–3.81)	0.977
Neoadjuvant treatment	0.89 (0.23–3.45)	0.868
Total ischemic time	1.94 (1.23–3.05)	0.004
Donor age	0.94 (0.89–0.99)	0.016
Male donor sex	0.89 (0.27–2.95)	0.855
Intraoperative PRBC transfusions (units)	1.14 (0.96–1.35)	0.127
Intraoperative FFP transfusions (units)	1.09 (0.92–1.30)	0.301

Hazard ratios are for quantitative variables were given per: 1 year increase for recipient and donor age; 1 point increase for MELD; 1 increase for tumor number; 1 cm increase for tumor size; 1 log_e_ increase for alpha-fetoprotein and Ca 19-9; 1 h increase for total ischemic time; 1 unit increase for transfusions. HR = hazard ratio; 95% CI = 95% confidence interval; MELD = model for end-stage liver disease; HBV = hepatitis B virus; HCV = hepatitis C virus; ALD = alcoholic liver disease; PRBC = packed red blood cells; FFP = fresh frozen plasma.

**Table 3:** Subgroup analyses of risk factors for tumor recurrence after liver transplantation in 18 patients without hepatitis B virus infection and 20 patients with intrahepatic tumor dissemination.

Factors	HBV-Negative (*n* = 18)		Intrahepatic Dissemination (*n* = 20)	
	HR (95% CI)	*p*	HR (95% CI)	*p*
Male sex	0.65 (0.06–6.42)	0.709	0.75 (0.15–3.84)	0.735
Age	1.06 (0.88–1.28)	0.516	1.01 (0.92–1.11)	0.813
MELD	1.11 (0.98–1.25)	0.098	1.02 (0.93–1.13)	0.621
HBV infection	-	-	2.85 (0.75–10.79)	0.123
HCV infection	1.04 (0.12–9.31)	0.975	1.09 (0.22–5.41)	0.918
ALD	1.88 (0.37–9.50)	0.444	0.65 (0.13–3.19)	0.598
AFP model	1.48 (1.01–2.17)	0.004	1.43 (1.06–1.92)	0.019
Tumor number	1.08 (0.83–1.41)	0.558	-	-
Tumor size	1.04 (0.56–1.91)	0.902	1.53 (1.01–2.30)	0.044
Alpha-fetoprotein	1.13 (0.86–1.49)	0.367	1.12 (0.91–1.38)	0.269
Ca 19-9	0.71 (0.13–4.01)	0.700	0.91 (0.37–2.20)	0.829
Poor tumor differentiation	1.83 (0.18–18.75)	0.612	3.19 (0.72–14.10)	0.127
Microvascular invasion	0.46 (0.07–3.09)	0.425	0.78 (0.21–2.95)	0.712
Macrovascular invasion	1.14 (0.16–8.11)	0.896	2.21 (0.53–9.31)	0.278
Neoadjuvant treatment	0.41 (0.05–3.61)	0.421	1.11 (0.28–4.43)	0.880
Total ischemic time	2.53 (1.19–5.37)	0.016	1.83 (1.12–2.98)	0.016
Donor age	0.90 (0.82–0.98)	0.017	0.94 (0.89–0.99)	0.041
Male donor sex	1.09 (0.22–5.42)	0.915	0.76 (0.22–2.67)	0.668
Intraoperative PRBC transfusions (units)	1.17 (0.94–1.47)	0.162	1.09 (0.91–1.30)	0.358
Intraoperative FFP transfusions (units)	1.07 (0.86–1.34)	0.548	1.05 (0.88–1.24)	0.619

Hazard ratios are for quantitative variables were given per: 1 year increase for recipient and donor age; 1 point increase for MELD; 1 increase for tumor number; 1 cm increase for tumor size; 1 log_e_ increase for alpha-fetoprotein and Ca 19-9; 1 h increase for total ischemic time; 1 unit increase for transfusions. HR = hazard ratio; 95% CI = 95% confidence interval; MELD = model for end-stage liver disease; HBV = hepatitis B virus; HCV = hepatitis C virus; ALD = alcoholic liver disease; PRBC = packed red blood cells; FFP = fresh frozen plasma.

**Table 4:** Analyses of risk factors for tumor recurrence after liver transplantation in 10 patients with macrovascular invasion included in the study.

Factors	HR (95% CI)	*p*
Male sex	0.41 (0.03–6.62)	0.533
Age	0.99 (0.88–1.11)	0.872
MELD	0.90 (0.67–1.20)	0.469
HBV infection	4.12 (0.57–29.66)	0.160
HCV infection	0.13 (0.01–1.46)	0.098
ALD	2.27 (0.20–25.32)	0.504
AFP model	1.14 (0.60–2.14)	0.694
Tumor number	1.24 (0.92–1.67)	0.154
Tumor size	0.86 (0.41–1.79)	0.688
Alpha-fetoprotein	0.93 (0.67–1.29)	0.654
Ca 19-9	0.66 (0.10–4.34)	0.663
Poor tumor differentiation	3.74 (0.22–64.64)	0.364
Neoadjuvant treatment	1.01 (0.10–10.29)	0.993
Total ischemic time	5.89 (0.68–51.18)	0.108
Donor age	0.96 (0.90–1.04)	0.346
Male donor sex	3.14 (0.32–30.55)	0.325
Intraoperative PRBC transfusions (units)	1.54 (0.97–2.44)	0.066
Intraoperative FFP transfusions (units)	1.20 (0.80–1.81)	0.374

Hazard ratios are for quantitative variables were given per: 1 year increase for recipient and donor age; 1 point increase for MELD; 1 increase for tumor number; 1 cm increase for tumor size; 1 log_e_ increase for alpha-fetoprotein and Ca 19-9; 1 h increase for total ischemic time; 1 unit increase for transfusions. HR = hazard ratio; 95% CI = 95% confidence interval; MELD = model for end-stage liver disease; HBV = hepatitis B virus; HCV = hepatitis C virus; ALD = alcoholic liver disease; PRBC = packed red blood cells; FFP = fresh frozen plasma.

## Data Availability

The datasets generated or analyzed during the current study are available from the corresponding author on reasonable request.
